# The Diagnosis of Cancer of the Uterus

**Published:** 1897-03-27

**Authors:** Walter C. Swayne

**Affiliations:** Obstetric Physician, Bristol Royal Infirmary; Lecturer on Practical Midwifery, Bristol Medical School.


### THE DIAGNOSIS OF CANCER OF THE UTERUS.

By Walter C. Swayne, M.D., London; Obstetric Physician, Bristol Royal Infirmary; Lecturer on Practical Midwifery, Bristol Medical School.

The successful treatment of malignant disease of any organ depends on its recognition at a stage of its development sufficiently early to give hope that its complete removal by surgical means is possible, when, possibly a permanent cure in the minority, or a latent period of greater or less length before recurrence, in the majority of cases, may be attained.

In the case of malignant disease of the uterus this is, perhaps, more true than in that of many other organs, since statistics show that recurrence is later as a rule in cases where the disease has been treated by ablation of the entire organ in the case of the uterus than in the case of the various other organs usually affected, and there is little doubt that earlier recognition would materially improve the results obtained.

The results hitherto obtained by the surgical ablation of the organ have in the hands of the best operators been most encouraging, the immediate mortality being smaller than that in operations on, say, the breast, while the latent period after operation is considerably longer than that obtained after operation for malignant disease on any other organ. In fact, at periods varying from two to seven years after operation, it has been found that the number of patients operated on and remaining healthy varies from 56 to 66 per cent. of the total number relating to each period, *i.e.*, that of patients examined who have been operated on four years previously 59·5 per cent. will be found to be free from recurrence, while of those operated on five years previously the same number, and of those operated on six years previously 66 per cent. will be found to be healthy. These figures, of course, refer to the number actually living and examined out of the total number operated on, but they seem to be fairly hopeful, and there is no doubt that earlier diagnosis and operation would materially add to the figures both as regards immediate success and delayed recurrence. The difficulties in the way of an early diagnosis are great, and can only be got over by careful attention to detail and the investigation, as a matter of routine, of any case where symptoms which might be produced by malignant disease are present.

It is quite impossible to make a diagnosis of this disease from symptoms only, and difficult to make it from symptoms and physical signs together, since many other affections produce symptoms and physical signs resembling those of malignant disease. Pain is a fairly constant but unreliable symptom; we meet with very advanced cases where it has never been present to a degree sufficient to alarm the sufferer. Fœtid discharge is equally characteristic of senile endometritis, sloughing polypus, or foreign bodies. A discharge of pus, however, from the uterus should cause further investigation, since this disease is one of the conditions which gives rise to it. Hæmorrhage, either in the form of menorrhagia or metrorrhagia, is common to numerous other diseases; hæmorrhage in a married woman after sexual intercourse is, however, a very suspicious sign, and is often one of the earliest symptoms of malignant disease. Enlargement of the fundus uteri may be due to benign growths, and is not characteristic, although a peculiar elastic and tense feeling is described as being present in cases of this disease. The characteristic enlargement is, however, eccentric, *i.e.*, it is characterised by enlargement of the cavity as well as of the fundus. Cachexia is not a sign of the least value, and is as often absent as not; if it has become marked the probability is that the disease has become well established and less likely to be benefited by removal. The presence of all of the classical symptoms renders the recognition of the condition easy; but when we remember that watery and fœtid discharge means sloughing and sepsis, that cachexia indicates toxæmia and anæmia, that pain, if characteristic is, caused by contraction of the uterus in order to expel growth, it is obvious that the presence of these symptoms will affect injuriously not only the success of the immediate operation but the prospect of recurrence being delayed, so that it is essential that the disease be recognised before the occurrence of these symptoms. We have, therefore to fall back upon what is the earliest symptom, namely, hæmorrhage, and it is only by careful consideration of the various causes of this that we can hope to arrive at a correct conclusion. The hæmorrhage of malignant disease is, as a rule, independent of pregnancy or parturition, although we find that not infrequently a woman previously healthy has one or two miscarriages before developing malignant disease, which are really due to the fact that the morbid process has already commenced. The complication that may arise on account of the possibility of the hæmorrhage being due to causes connected with child bearing are, of course, absent in the sterile and the virgin, neither of whom are in any way exempt from the affection, and also after the menopause, when this symptom becomes of the gravest import; not only does it call for a careful examination in women previously healthy, but it is a sign that a pre-existing benign growth is taking on malignant changes which are apt to progress with great rapidity.

The earliest symptom then is hæmorrhage in the form of a gradually increasing menorrhagia; this, if we can exclude personal habits, *e.g.*, alcoholism, Bright's disease, &c., should lead to a careful exploration, when, if enlargment of the body of the uterus is found not having the peculiar nodulated feel of a myoma, the cavity should be explored with the finger after dilatation of the cervix, when possibly a polypus may be discovered; if, however, this is not the case, and the cavity contains any mass of a polypoid-form or merely a condition of fungating mucous membrane, several as large pieces as possible should be removed for microscopic examination, which, if confirming suspicions already awakened, should lead to ablation of the organ. Even with these precautions, a certain number of cases will be passed over. While the female mind, as a whole, refuses to recognise that menstrual irregularity in the way of excess is anything that may be of importance medically, while hæmorrhage after the menopause is hailed as the "return of nature," instead of a danger signal of the most unmistakable kind, cases of malignant disease will be allowed to progress until the more unmistakable symptoms are present, and both the immediate mortality and length of time without recurrence will be influenced unfavourably.

Cancer affecting the cervix uteri is recognised with much greater ease on account of its greater accessibility; but, at the same time, many cases only come for medical assistance when the disease has become so extensive as to preclude any operative interference. The same symptoms are of importance here as in the affection of the body, and the chief here, too, is hæmorrhage, and notably hæmorrhage following sexual intercourse or digital examination. The conditions most likely to cause confusion in cases suffering from hæmorrhages before the menopause are fibroid myoma, polypi, and endometritis fungosa.

The enlargement of the uterus caused by myoma is not usually symmetrical, and is usually nodular or lobulated. Polypi, if not presenting at the os, are easily discovered after dilatation of the cervix; both will usually present a history of sterility of some duration; endometritis cannot be distinguished from a case of malignant disease in its early stage, except by the fact that the enlargement of the fundus is not progressive, and is combined with symptoms of inflammatory affection of the cervix and vaginal portion.

After the menopause the condition which most simulates carcinoma is senile endometritis; here the fœtid discharge, and sometimes occasional hæmorrhage, render the resemblance very striking. Dilatation of the cervix and exploration of the cavity will reveal the absence of marked enlargement or new growth, and after curetting and thorough irrigation a permanent cure will be established.

There can be no question but that hæmorrhage in the form of menorrhagia or metrorrhagia is a sign that should be regarded as of more importance than it has been hitherto. Provided that in any case of hæmorrhage we can exclude the more obvious and simple causes, we should never loose sight of the fact that the onset of malignant disease of the body of the uterus is most insidious, and that its successful surgical treatment depends entirely on its early discovery. The mortality of the operation by which the uterus is removed is not large; it averages about 8 per cent., and varies from 4·5 to 11 or 12 in the hands of various individual operators. This operation, vaginal hysterectomy, is proving far more successful than any of the other methods of removing the uterus; it is, indeed, the only method suitable to the treatment of cases of cancer of the body of the uterus, while it is probably also the best method of dealing with cancer of the cervix. The following are fairly typical cases of this disease.

Patient aged 45, multipara, sought advice on account of pain in the back with menorrhagia, which had been getting more and more profuse. She had never been pregnant. On examination there was found to be a considerable amount of discharge from the cervix, no hæmorrhage, and no fœtor. The fundus was felt to be enlarged, slightly tender, and not displaced. The sound passed three and a half inches, and caused slight sanguineous discharge. She was advised to undergo exploration of the uterus, to which she agreed. The cervix was dilated, and on exploring the uterine cavity a friable mass was felt on the posterior wall; a quantity of this was removed, and on microscopical examination proved to be carcinoma. Vaginal hysterectomy was performed, and the patient made a good recovery and remains well after two years.

Another case was that of a nullipara, aged 41. She had miscarried 18 months before seeking advice, which she did on account of increasing menstrual flow, and also of free discharge, which occurred between the periods, and which latterly had been offensive; the uterus was markedly enlarged, and its mobility impaired. Exploration was advised and agreed to. A polypoid growth was found on the anterior wall of the uterus, very soft and friable, and partly breaking down. Several portions were removed, and were found to consist of malignant growth. Vaginal hysterectomy was performed, and the patient made a good recovery.

In the first of these two cases the possibility of the patient having malignant disease would not have been considered but for the slight sanguineous discharge on passing the sound, previous to which it was thought that endometritis, with possibly a polypus, might be the cause. The dilatation and exploration, however, at once cleared up all doubts, while the microscope gave confirmatory evidence.

In the second case the symptoms were more suspicious, but no certainty could be felt until after dilatation and curetting proved the existence of the growth. Hæmorrhage is undoubtedly the earliest symptom of cancer of the body of the uterus, and if it is found that some simple or benign cause does not produce it, investigations to prove the absence of malignant disease should be made. Should it be proved that a malignant growth is present, its operative treatment may be undertaken with every hope of a good result, and, while menorrhagia may be caused by several simple conditions, metrorrhagia is, in the absence of fibroid tumour or polypus, always a sign which should lead to the viewing of the case with suspicion.

